# Characteristics of the True Believer in Later Life

**DOI:** 10.1093/geroni/igaa057.2256

**Published:** 2020-12-16

**Authors:** Shannon Jones, Austin Prusak, Rennae Wigton, Andrew Futterman

**Affiliations:** Loyola University Maryland, Baltimore, Maryland, United States

## Abstract

This study describes the nature, causes, and outcomes of steadfast, unquestioned “true” belief in later life (Hoffer, 1950). According to some theorists, such “true” belief develops from more childish, extrinsically-motivated, compartmentalized beliefs and behaviors to become more mature, intrinsically-motivated, comprehensive and integrated beliefs and behaviors (e.g., Allport, 1950). Research, however, is equivocal regarding the validity of this position. From “grounded-theory” analysis of a sample of 278 semi-structured interviews of older adults from six New England states and New York (aged 55-101 years-old.), we demonstrate the need for a more nuanced definition of a “true” belief as a form of religiousness without commitment to rigid orthodoxy. For example, a sizable segment of this sample changed religious denominations over the course of their lives without ever doubting the presence of a diety (i.e., a God, a Higher Power, etc.), but who dramatically changed the way they expressed this belief. This prompts a reconceptualization of “true” belief by Hoffer and a more nuanced understanding of religious development than implied by Allport, one that more adequately accounts for individual differences in life experiences, personality, religious upbringing, and religious cultural expectations. We discuss these findings in light of recent research by Wink and Dillon (2002, 2008).

